# Supplementary material for: Evolution of linear triterpenoid biosynthesis within the Euphorbia genus
Source: Nat Commun. 2025 Jul 1;16:5602. doi: 10.1038/s41467-025-60708-2 (PMC12219392; doi:10.1038/s41467-025-60708-2)
Supplement: Supplementary file 1 — Supplementary Information [file 41467_2025_60708_MOESM1_ESM.pdf]

Evolution of linear triterpenoid biosynthesis within the *Euphorbia* plant genus

Czechowski *et al.*

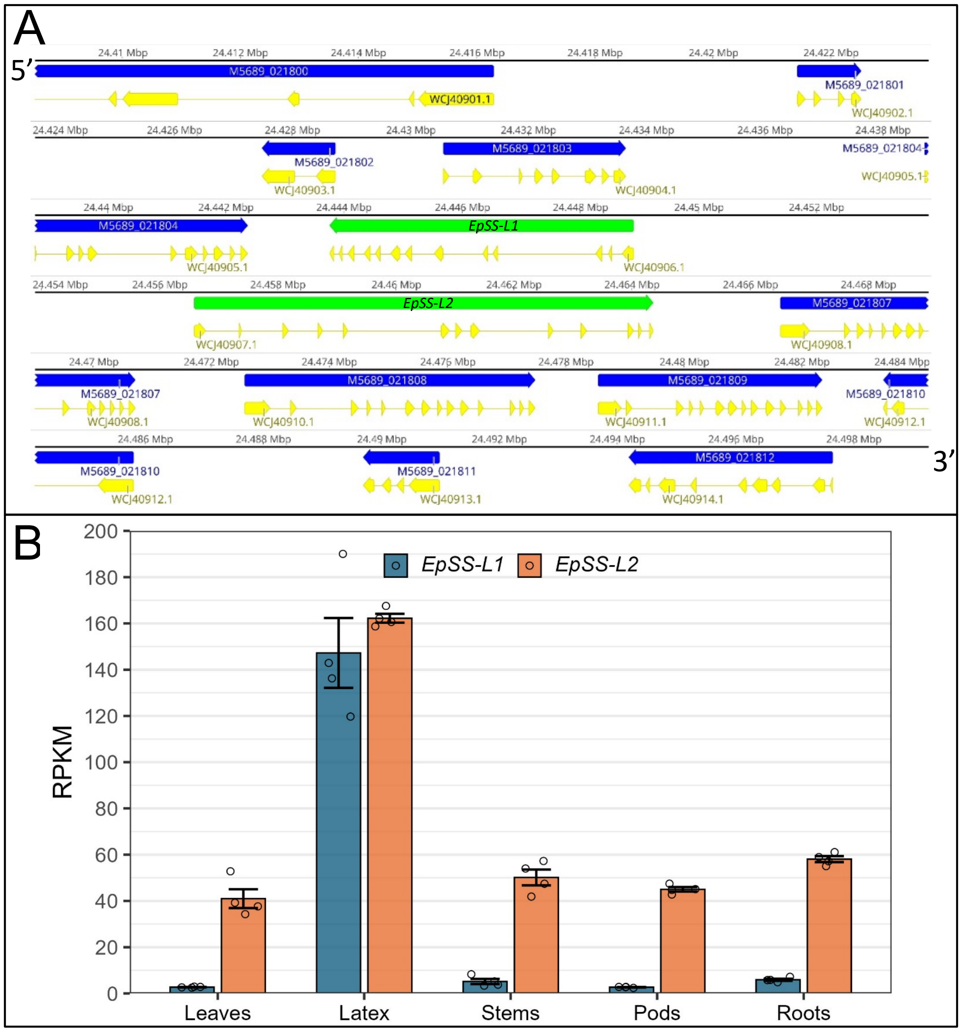

Supplementary Figure 1. Genomic location and expression profile of two putative squalene synthases from *E. peplus.* (A). Location of putative squalene synthase genes on chromosome 7 of *E. peplus* genome assembly from [1]. Blue/green tracks – genes, yellow tracks – CDS **(B).** Expression profiling of putative squalene synthases in five *Euphorbia peplus* tissues. RPKM - Reads Per Kilobase of transcript per Million mapped reads. Error bars – SEM (n=4, where n is the number of biological replications). Source data are provided as a Source Data file.

A

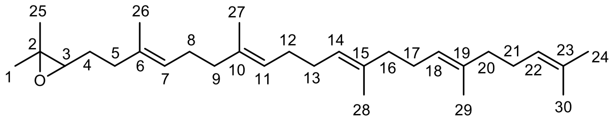

2,3-Oxidosqualene

B

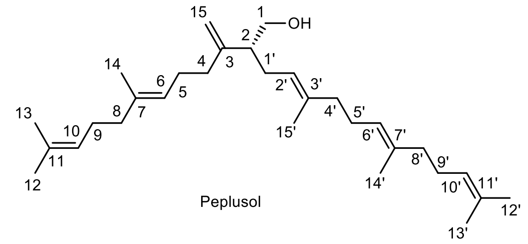

Supplementary Figure 2. NMR-characterized structures of 2,3-oxidosqualene and peplusol. Structures presented for *N. benthamiana* - extracted and purified 2,3-oxidosqualene **(A)** and *E. peplus* and *E. lateriflora* - extracted and purified peplusol **(B).**

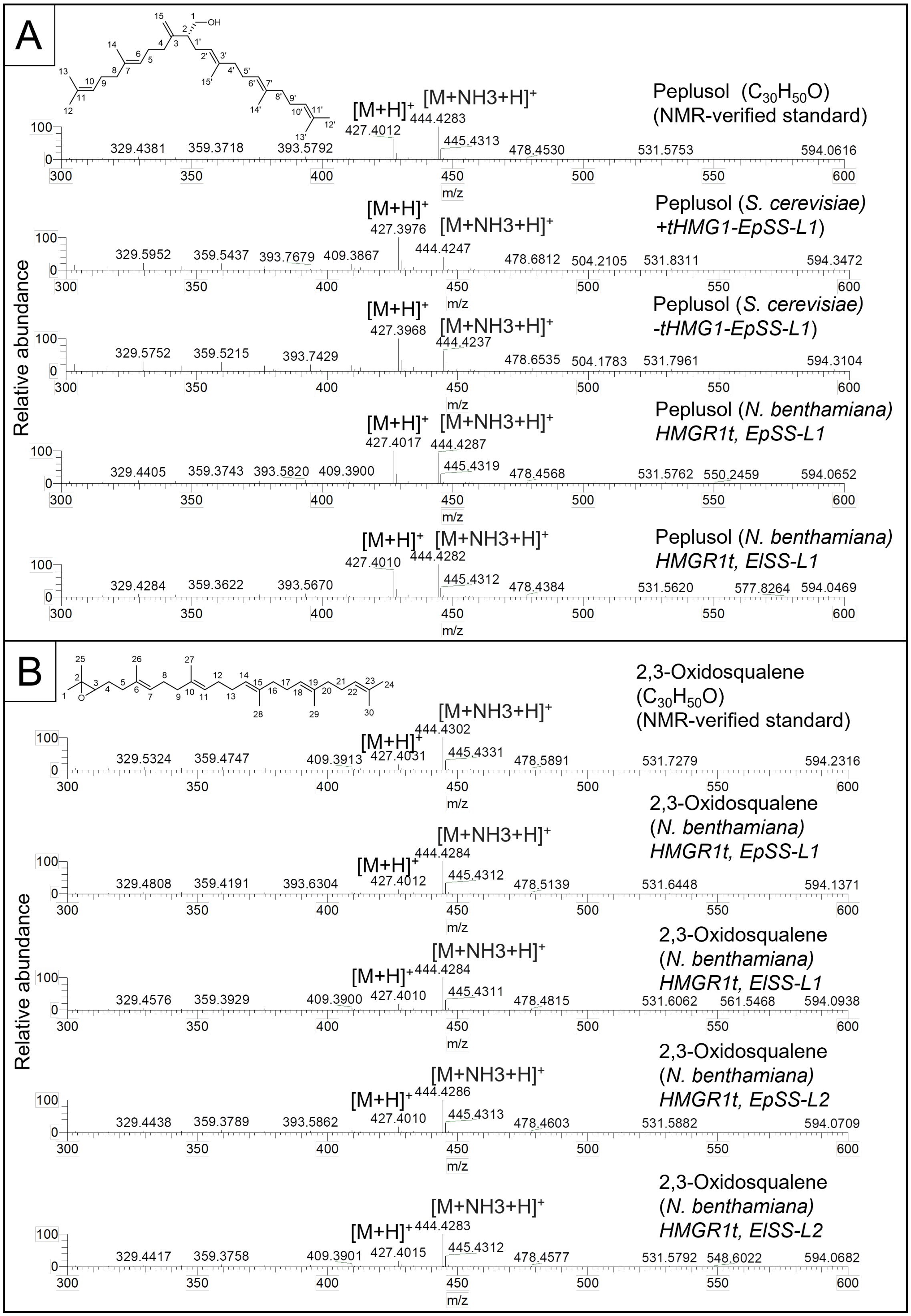
A
Peplusol (C30H50O)
(NMR-verified standard)
[M+H]+
427.4012
[M+NH3+H]+
444.4283
Peplusol (S. cerevisiae)
+tHMG1-EpSS-L1)
Peplusol (S. cerevisiae)
-tHMG1-EpSS-L1)
Peplusol (N. benthamiana)
HMGR1t, EpSS-L1
Peplusol (N. benthamiana)
HMGR1t, ElSS-L1
Relative abundance
m/z
B
2,3-Oxidosqualene
(C30H50O)
(NMR-verified standard)
2,3-Oxidosqualene
(N. benthamiana)
HMGR1t, EpSS-L1
2,3-Oxidosqualene
(N. benthamiana)
HMGR1t, ElSS-L1
2,3-Oxidosqualene
(N. benthamiana)
HMGR1t, EpSS-L2
2,3-Oxidosqualene
(N. benthamiana)
HMGR1t, ElSS-L2

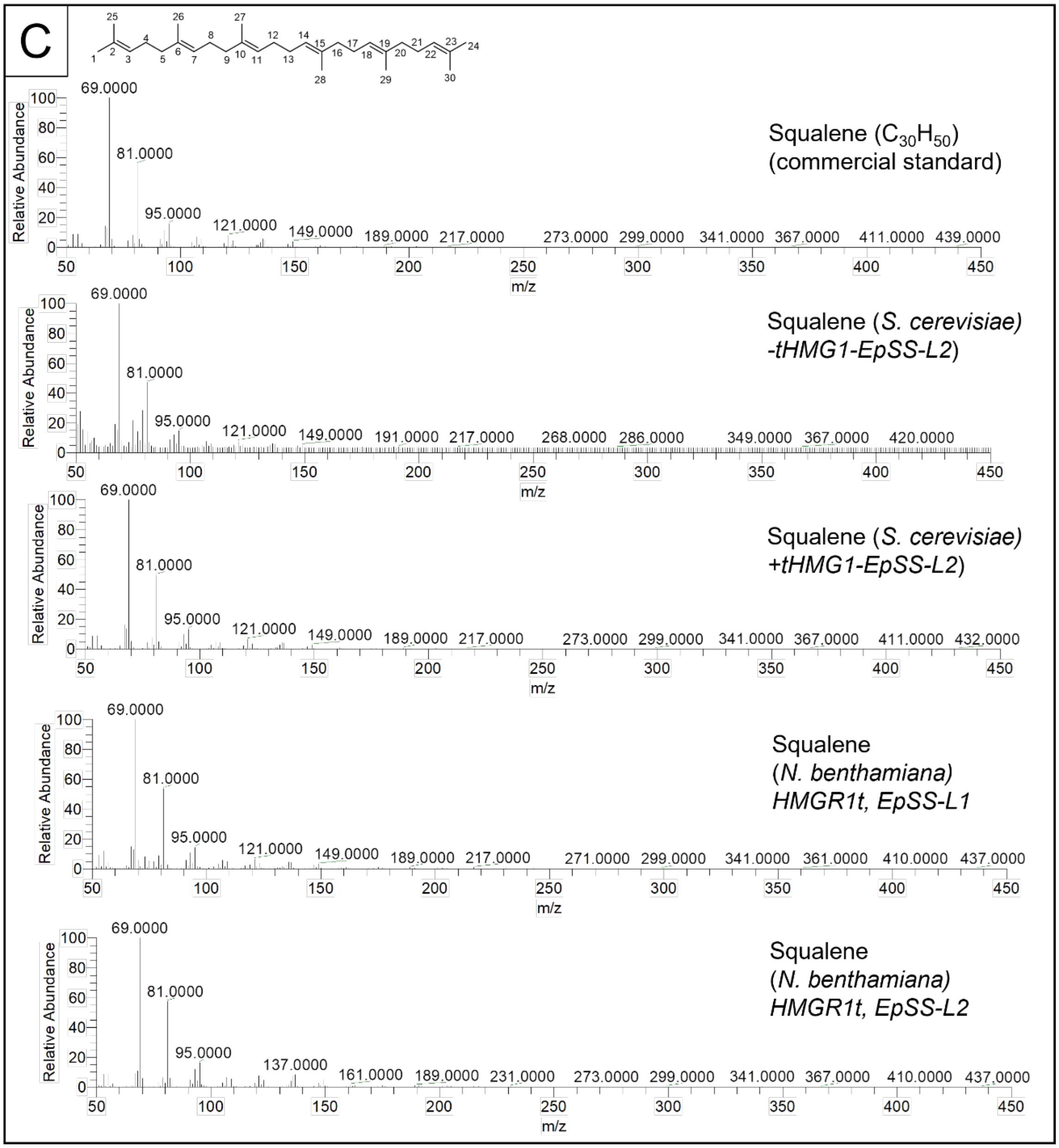

Supplementary Figure 3. LC- and GC-MS derived MS data for peplusol, 2,3-oxidosqualene and squalene. HMRS data for samples extracted from *N. benthamiana* as well as NMR-verified standards for peplusol **(A)** and 2,3-oxidosqualene **(B).** GC-MS derived *m/z* profiles of squalene extracted from *N. benthamiana* and *S cerevisiae* samples as well as those for commercial squalene standard **(C)**.

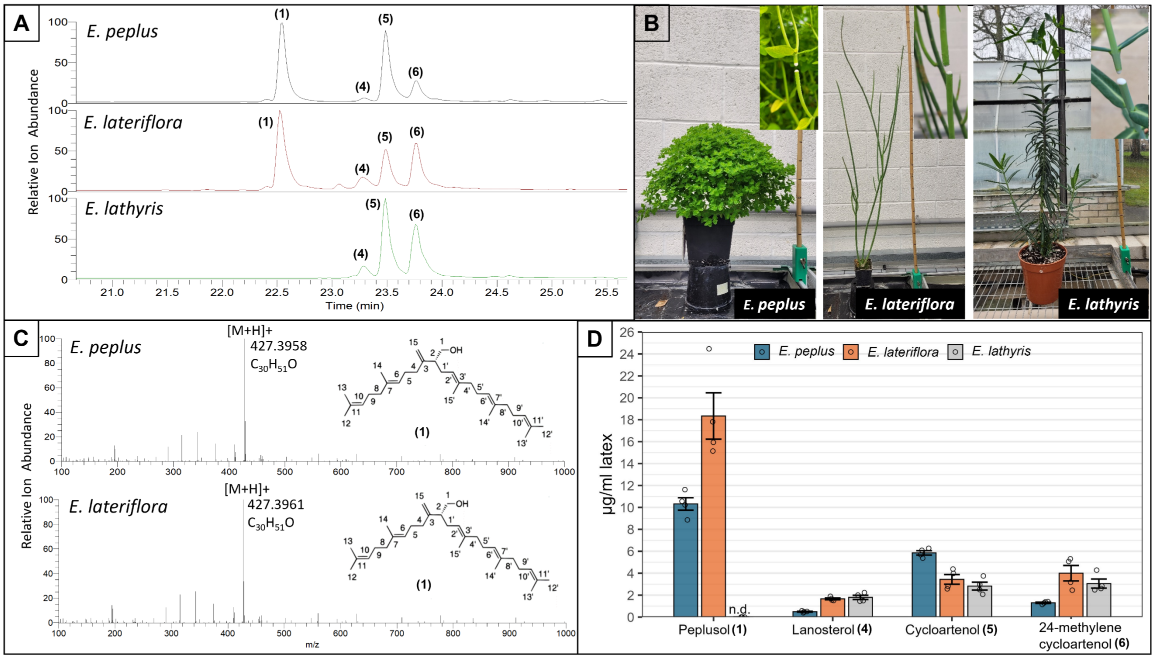

Supplementary Figure 4. Metabolite profiling of *E. peplus, E. lateriflora* and *E. lathyris* latex. (A) Total Ion Current (TIC) chromatograms shown for each of the three species with four major latex triterpenoids annotated: peplusol **(1)**, lanosterol **(4)**, cycloartenol **(5)** and 24-methylenecycloartenol **(6)**. **(B)** Plant morphology of *E. peplus, E. lateriflora* and *E. lathyris* at the time latex was harvested. **(C)** HRMS data for peplusol (**1**) from both species together with NMR-verified structures of peplusol isolated and purified from *E. peplus* and *E. lateriflora*. **(D)** Quantitative analysis of four major triterpenoids presented on panel A in latex tissues of *E. peplus, E. lateriflora* and *E. lathyris*. Metabolites extracted and quantified via LC-MS as described in methods. Error bars - SEM (n=4, where n is the number of biological replications). n.d.: not detectable. Source data are provided as a Source Data file.

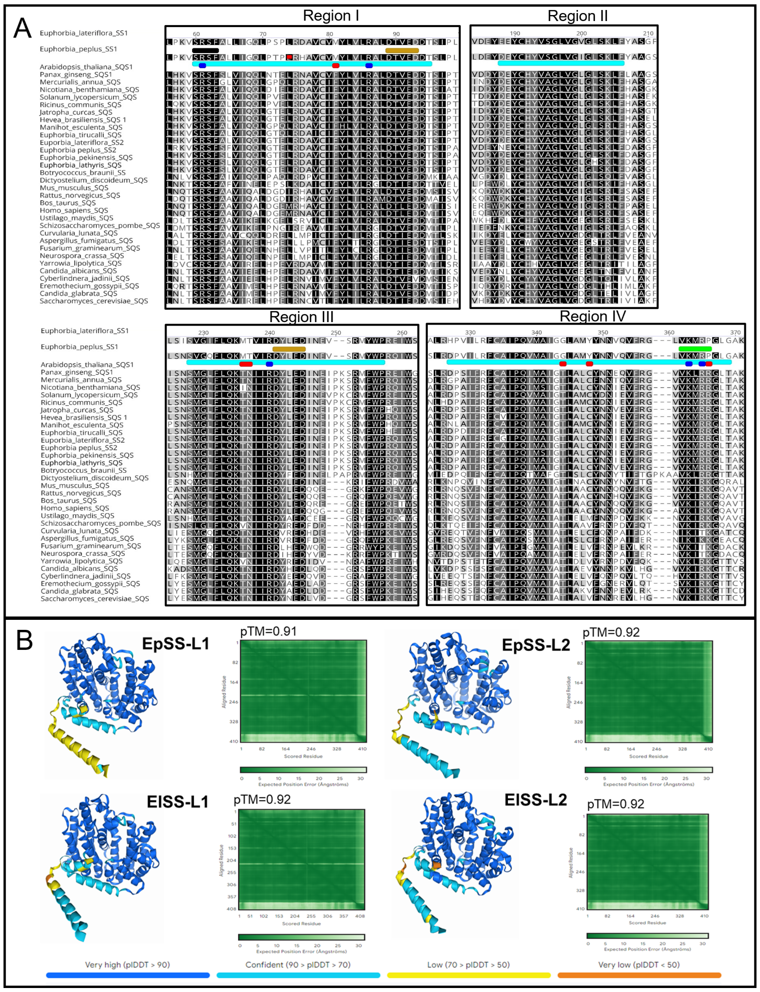

Supplementary Figure 5. Identification of amino acid positions involved in peplusol synthase activity and *in silico* protein structure modelling using the Alpha Fold 3 server. (A) MUSCLE alignment and of cDNA-predicted amino acid sequences and phylogenetic tree built for fifty functionally characterised squalene synthases from plant, animal and fungi with functionally characterised *E. peplus* and *E. lateriflora* peplusol synthases: EpSS-L1 and ElSS-L1. Alignment shown only for four conserved regions (highlighted in light blue) previously shown to confer squalene synthase activity [2]. Two DXXED motifs involved in the FPP substrate binding sites are highlighted in brown. Two mobile segments: the AB flap, assumed to regulate the binding of substrates (prenyl donor and acceptor) and JK loop, crucial for NADPH binding [2], highlighted in black and green, respectively. Residues involved in NADPH recognition are highlighted in deep blue and the seven positions that are unique for EpSS-L1 and ElSS-L1 and conserved in squalene synthases in red. (**B)** cDNA-predicted amino acid sequences of EpSS-L1, EpSS-L2, ElSS-L1 and ElSS-L2 were used to model 3D protein structure using Alpha Fold 3 [3]. Modelling confidence shown for each of the four models as: a) pLDDT a per-atom confidence estimate on a 0-100 scale where a higher value indicates higher confidence, as indicated by colours on the structure, b) PAE (predicted aligned error): estimate of the error in the relative position and orientation between two tokens in the predicted structure. Higher values indicate higher predicted error and therefore lower confidence and c) template modelling (pTM) score which measures the accuracy of the entire structure on a scale 0-1.

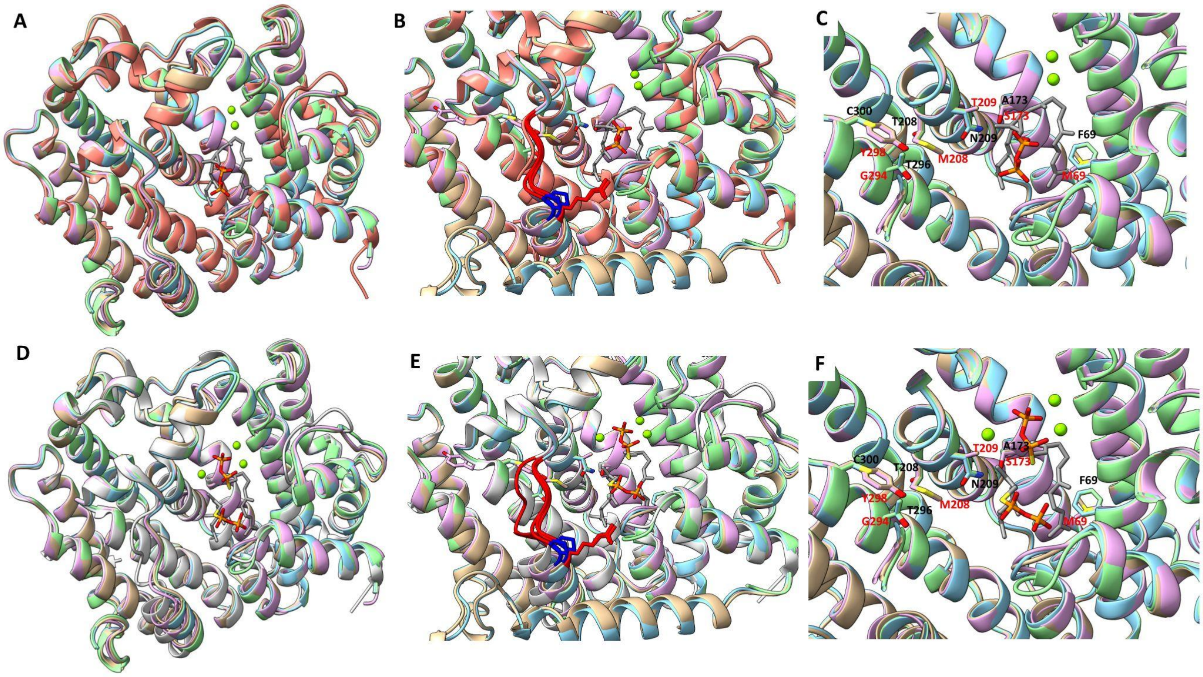

Supplementary Figure 6. Structural overlays of AlphaFold3 models for EpSS-L1, EpSS-L2, ElSS-L1 and ElSS-L2 with human Squalene Synthase protein crystal structures. cDNA-predicted amino acid sequences of EpSS-L1,EpSS-L2, ElSS-L1 and ElSS-L2 were used to model 3D protein structure using Alpha Fold 3 [3]. Highest ranked models for EpSS-L1 (magenta), EpSS-L2 (green), ElSS-L1 (brown) and ElSS-L2 (blue) were overlaid with: **(A-C)** PDB-deposited (3weh, red) structure human squalene synthase in complexes with presqualene pyrophosphate (PSPP, grey) and Mg2+ (green spheres) or **(D-F)** PDB-deposited (3weg, grey) structure human squalene synthase in complexes with farnesyl thiopyrophosphate (FsPP, a non-cleavable FPP analogue, grey) and Mg2+ (green spheres). Structural overlays were created using UCSF ChimeraX v 1.7 [4] using the 3weh (A-C) or 3weh (D-F) model as a reference, as described in methods. Flexible loop crucial for NADPH binding by human squalene synthase highlighted in red with R315 conserved for EpSS-L2, ElSS-L2 (red) and P313 conserved for EpSS-L1, ElSS-L1 (blue) shown as stick and wires on panels B and E. 6Å region around PSPP (C) and FsPP (F) binding site shown with amino acids identified as potentially important for peplusol synthase activity displayed as stick and wires and labelled for EpSS-L1, ElSS-L1 in red and for EpSS-L2, ElSS-L2 in black.

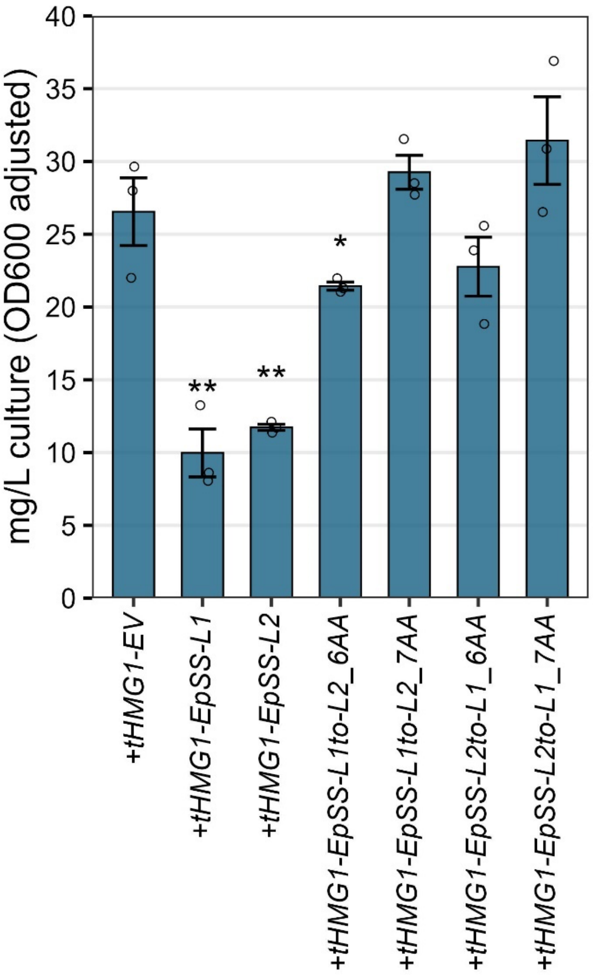

Supplementary Figure 7. Squalene content for *EpSS-L2* and *EpSS-L1* hexa- and heptamutants expressed in *S. cerevisiae truncated* HMG-CoA reductase 1 - expressing strain (+*tHMG1*). *S. cerevisiae truncated HMG-CoA reductase 1* -expressing strain (+tHMG1) was transformed with either: Empty Vector (EV, pBEVY-L) or pBEVY-L constructs for overexpression of: *E. peplus Squalene Synthase-like2* (*EpSS-L2*, GenBank locus tag M5689_021806), *E. peplus Squalene Synthase-like2 hexaswap* (*EpSS-L2to-L1_6AA*: F69M, T208M, N209T, T296G, C300Y and K318P), *E. peplus Squalene Synthase-like2 heptaswap* (*EpSS-L2to-L1_7AA*: F69M, T208M, N209T, T296G, C300Y, K318P and A173S), *E. peplus Squalene Synthase-like1* (*EpSS-L1*, GenBank locus tag M5689_021805), *E. peplus Squalene Synthase-like1 hexaswap* (*EpSS-L1to-L2*_6AA: M69F, M208T, T209N, G294T, Y298C, P313R) and *E. peplus Squalene Synthase-like1 heptaswap (EpSS-L1to-L2*_7AA: M69F, M208T, T209N, G294T, Y298C, P313R and S173A). Three independent transformants were grown in liquid cultures. Squalene levels were quantified by GC-MS in cell pellets as described in methods. Error bars – SEM (n=3, where n is the number of biological replications). Statistically significant (one-sided *t*-test) differences between control (EV) and candidate genes indicated by asterisks (*: *p*-value <0.05, **: *p*-value <0.01). Source data are provided as a Source Data file.

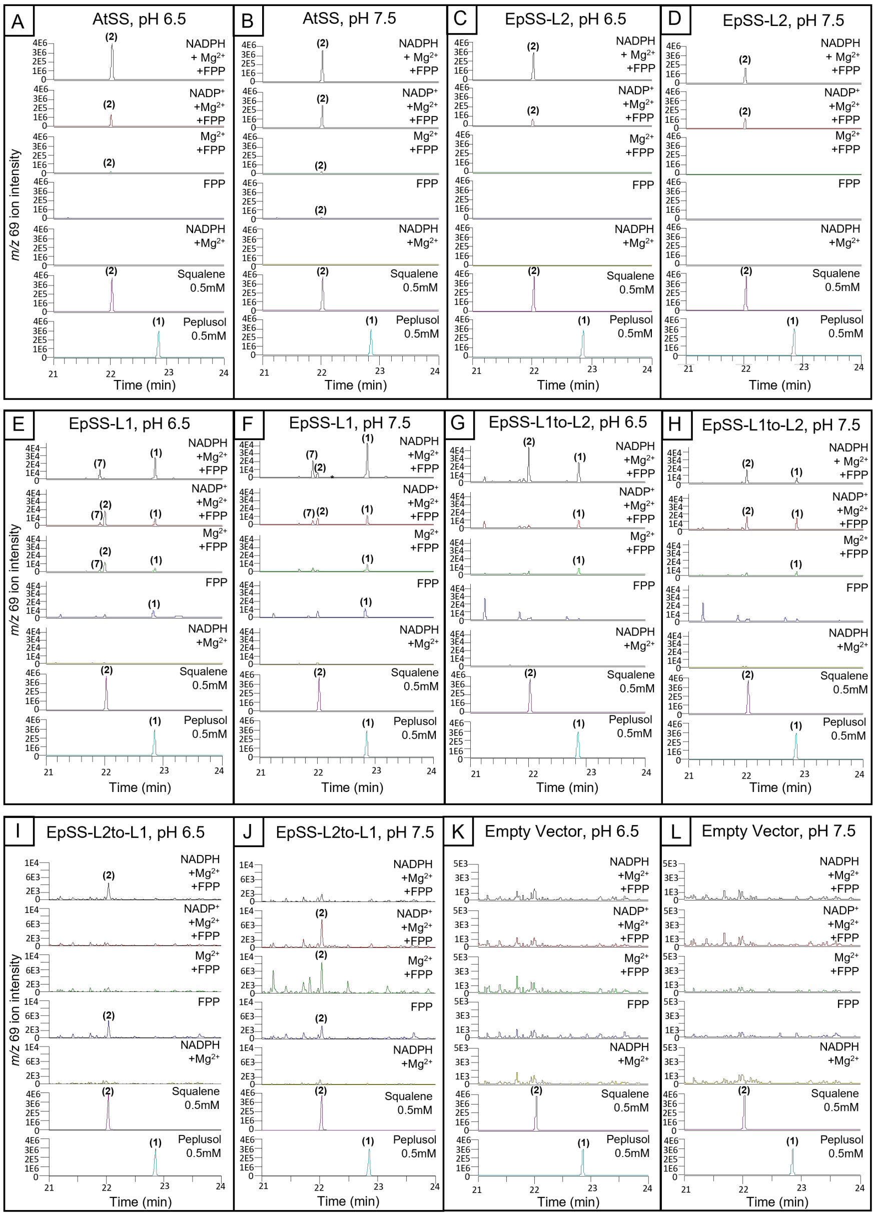

Supplementary Figure 8. Results of the *in vitro* enzyme activity assays for AtSS, EpSS-L1, EpSS-L2, EpSS-L1to-L2 and EpSS-L2to-L1 heptaswap mutants. *In vitro* enzymatic reactions were conducted using total protein lysates from *E. coli* strains expressing: **(A,B)** *A. thaliana Squalene Synthase (AtSS),* **(C,D)** *E. peplus Squalene Synthase (EpSS-L2),* **(E,F)** *E. peplus Peplusol Synthase (EpSS-L1),* **(G,H)** *E. peplus Peplusol- to Squalene Synthase heptaswap (EpSS-L1to-L2),* **(I,J)** *E. peplus Squalene- to Peplusol Synthase heptaswap (EpSS-L2to-L1)* as well as strains transformed with the empty vector **(K,L)**. Various co-factors of the reactions including NADPH, $NADP^{+}$, Mg^{2+} were tested as described in methods section. Hexane/EthylAcetate extracted and derivatized products of enzymatic assays were analyzed by GC-MS in parallel with *E. peplus* latex-purified peplusol and commercial squalene standards. Extracted Ion Chromatograms (EIC) for *m/z* 69 showing peplusol (**1**), squalene (**2**), and un-identified compound (**7**).

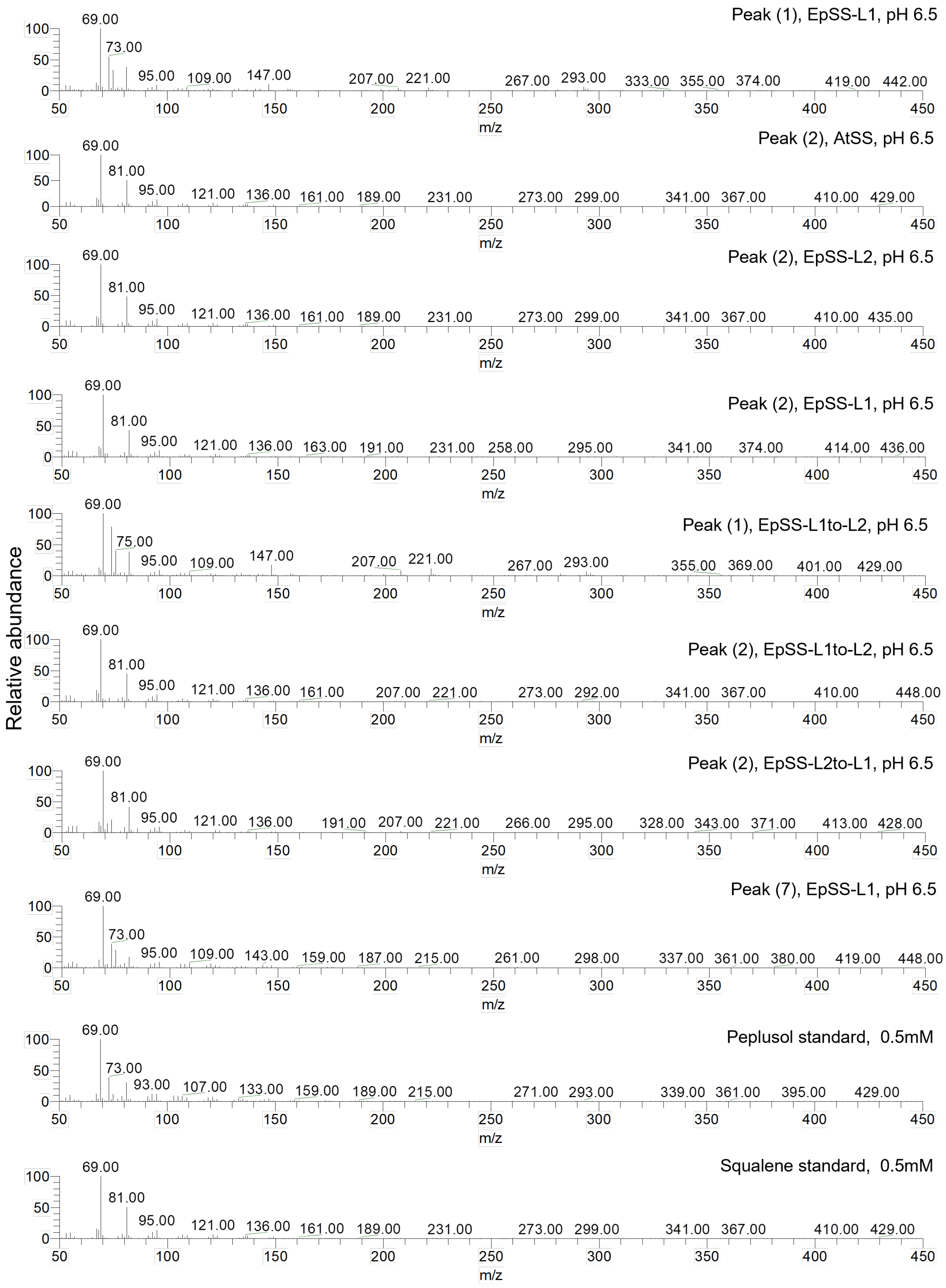

Supplementary Figure 9. GC-MS derived *m/z* profiles of the products of the selected *in vitro* enzymatic reaction as well as those for peplusol and squalene standards.

Supplementary Table 1. Primers used for COS analysis of *Euphorbia lathyris* plant material.

Gene	*E. lathyris* genomic coordinates	*A. thaliana* homologue	Gene annotation	Forward (5'-3')	Reverse (5'-3')
Agt1	OY755225.1:14453648-14458991	AT2G13360	alanine:glyoxylate aminotransferase	GGCATTAACCGGTTCACAG	CCTCATTGAGATTGCCAAGATG
AroB	OY755229.1:c4143650-4139650	AT5G66120	3-dehydroquinate synthase	GTTTGATAAAGCTATTGGATCACG	TCAAAAAATTCAGCATCTCTTATAAGC
At103	OY755228.1:c73927075-73921903	AT3G56940	putative dicarboxylate diiron protein	TTGACAAAGGCTAGAAAATACACA	CAATCATTGAGGTACATTGTCAC
gbssi	OY755220.1:125705500-125709950	AT1G32900	granule bound starch synthase 1	CAGGGTTTTGACTGTGAGC	GGAACCCTGTGTAACCTTCT

Supplementary Table 2. Primers used for cloning and sub cloning of *E. peplus* and *E. lateriflora* candidate gene cDNAs into pEAQ-HT (*N. benthamiana* transient expression vector) with corresponding GenBank accession numbers.

Gene ID	GenBank Accession	SwissProt annotation	Forward (5'--3')	Reverse (5'--3')
EpSS-L1	WCJ40906.1	Squalene synthase-like	CTGTATATTCTGCCCAAATTCGCGAAAAAATGGAGATTTTGGGAGGGATAG	AATTTAATGAAACCAGAGTTAAAGGTCAGTAAGTGGTCATTTTTTTGTG
EpSS-L2	WCJ40907.1	Squalene synthase-like	CTGTATATTCTGCCCAAATTCGCGAAAAAATGGGGAGTTTGGGAGC	AATTTAATGAAACCAGAGTTAAAGGTTAGTTGGTCCGATTGCAG
ElSS-L1	PP978604	Squalene synthase-like	CTGTATATTCTGCCCAAATTCGCGAAAAAATGGAGATTTTGGGTGGAATAGTG	AATTTAATGAAACCAGAGTTAAAGGTTAGTAAGTGGTCATTTTTTTGAGATATGC
ElSS2-L2	PP978605	Squalene synthase-like	CTGTATATTCTGCCCAAATTCGCGAAAAAATGGGGAGTTTGGGAGC	AATTTAATGAAACCAGAGTTAAAGGCTAGTTGGTCCGATTGCC

Supplementary Table 3. Primers used to clone guide sequence (in red) and to amplify linear DNA parts from CEN.PK2-1C strain.

pCASsgRNA_ARS911-F	CGGGTGGCGAATGGGACTTTGTAATATTGTCTTGTTTCCCGTTTTAGAGCTAGAAATAGC
pCASsgRNA_ARS911-R	GCTATTTCTAGCTCTAAAACGGGAAACAAGACAATATTACAAAGTCCCATTCGCCACCCG
PTDH3-ARS911overlap_F	ATAGAAAAAAATCGGATGTTGAATGGGCATAAATATAAATGTATATATAAGAATAAAAAACACGCTTTTTCAGTTCG
PTDH3-tHMG1overlap_R	TCTTCACCAATTGGTCTGCAGCCATTTTGTTTGTTTATGTGTGTTTATTCG
tHMG1-PTDH3overlap_F	GAATAAACACACATAAACAAACAAAATGGCTGCAGACCAATTGGTG
tHMG1-TCYC1overlap_R	TCCTTCCTTTTCGGTTAGAGCGGATTTAGGATTTAATGCAGGTGACGG
TCYC1-tHMG1overlap_F	GTCCGTCACCTGCATTAAATCCTAAATCCGCTCTAACCGAAAAGGAAG
TCYC1-ARS911overlap_R	TTTAAATTTTATGTCTGTTTTGTATGCTATTTCATTTTTCATTTACTTCTCTTCGAGCGTCCCAAAACCTTC

Supplementary Note 1. NMR data for 2,3-oxidosqualene and peplusol

NMR data for *N. benthamiana* - extracted 2,3-oxidosqualene: ^{1}H NMR (600 MHz, CDCl 3): δ 5.12 (m, 5H (H-7, H-11, H-14, H-18, H-22)), 2.71 (t, 1H (H-3)), 2.18-1.94 (m, 18H (H-5, H-8, H-9, H-12, H-13, H-16, H-17, H-20,H-21)), 1.70-1.55 (m, 2H (H-4)), 1.68 (s, 3H (H-24)), 1.62 (s, 3H (H-26)), 1.60 (s, 12H (H-27, H-28, H-29, H-30)), 1.26 (s, 3H (H-25)); 13 C NMR (151 MHz, CDCl 3): δ 135.1 (C-19), 135.0 (C-10/C-15), 134.9 (C-10/C-15), 134.0 (C-6), 131.3 (C-23), 125.0 (C-7), 124.4 (C-22), 124.3 (C-11, C-14, C-18), 64.3 (C-3), 58.4 (C-2), 39.8, 39.7(C-16, C-20, C-9), 36.3 (C-5), 28.3 (C-12, C-13), 27.5 (C-4), 26.8 (C-21), 26.7 (C-8'), 25.7 (C-24), 24.9 (C-1), 18.7 (C-25), 17.7 (C-30), 16.1, 16.0 (C-26, C-27, C-28, C-29).

HRMS (*m/z*) [M+H]+ calculated for $C_{30}H_{50}O$, 427.3940; found, 427.4031. NMR and HRMS data for 2,3-oxidosqualene are in agreement with previously published data[5,6]

NMR data for *E. peplus* and *E. lateriflora* - extracted peplusol (exact match): ^{1}H NMR (600 MHz, CDCl$_3$): δ 5.14-5.06 (m, 5H (H-6, H-10, H-2', H-6', H-10')), 4.96 (s, 1H (H-15)), 4.87 (s, 1H (H-15)), 3.59-3.51 (m, 2H (H-1)), 2.28 (ddt, J = 7.2, 5.5, 7.2 Hz, 1H (H-2)), 2.19-2.12 and 2.12-2.01 (m, (H-4, H-5, H-1', H-5', H-9')), 2.02-1.95 (m, 6H (H-8, H-4', H-8')), 1.68 (s, 6H (H-13, H-13')), 1.61 (s, 6H (H-12, H-12')), 1.60 (s, 6H (H-14', H-14 or H-15')), 1.59 (s, 3H (H-14 or H-15')); ^{13}C NMR (CDCl$_3$): δ 149.6 (C-3), 136.5, 135.6, 135.0, 131.4, 131.3 (C-7, C-11, C-3', C-7', C-11'), 124.4, 124.3, 124.1, 123.9 (C-6, C-10, C-6', C-10'), 122.2 (C-2'), 111.0 (C-15), 64.0 (C-1), 48.8 (C-2), 39.8, 39.7, 39.7 (C-8, C-4', C-8'), 34.4 (C-4), 29.1 (C-1'), 26.8, 26.7, 26.6 (C-5, C-9, C-9'), 26.2 (C-5'), 25.7 (C-13, C-13'), 17.7 (C-12, C-12'), 16.2, 16.1, 16.0 (C-14, C-14', C-15').

HRMS (*m/z*) [M+H]+ calculated for $C_{30}H_{50}O$, 427.3940; found, 427.4012. NMR and HRMS data for peplusol are in agreement with previously published data[7]

Supplementary references

1 Johnson, A. R. *et al.* Chromosome-level genome assembly of *Euphorbia peplus*, a model system for plant latex, reveals that relative lack of Ty3 transposons contributed to its small genome size. *Genome Biol Evol* **15**, evad018 (2023).

2 Liu, H. *et al.* Molecular digitization of a botanical garden: high-depth whole-genome sequencing of 689 vascular plant species from the Ruili Botanical Garden. *Gigascience* **8**, giz007 (2019).

3 Abramson, J. *et al.* Accurate structure prediction of biomolecular interactions with AlphaFold 3. *Nature* **630**, 493-500 (2024).

4 Meng, E. C. *et al.* UCSF ChimeraX: Tools for structure building and analysis. *Protein Sci* **32**, e4792 (2023).

5 Abad, J. L., Casas, J., Sanchezbaeza, F. & Messeguer, A. Dioxidosqualenes: Characterization and activity as inhibitors of 2,3-oxidosqualene lanosterol cyclase. *J Org Chem* **58**, 3991-3997 (1993).

6 Tian, Y., Xu, X., Zhang, L. & Qu, J. Tetraphenylphosphonium tetrafluoroborate/1,111,313,3-hexafluoroisopropanol (PhPBF/HFIP) effecting epoxide-initiated cation-olefin polycyclizations. *Organic Letters* **18**, 268-271 (2016)

7 Giner, J. L., Berkowitz, J. D. & Andersson, T. Nonpolar components of the latex of *Euphorbia peplus*. *J Nat Prod* **63**, 267-269 (2000).
