# Supplementary material for: Evolution of linear triterpenoid biosynthesis within the Euphorbia genus
Source: Nat Commun. 2025 Jul 1;16:5602. doi: 10.1038/s41467-025-60708-2 (PMC12219392; doi:10.1038/s41467-025-60708-2)
Supplement: Supplementary file 2 — Description of Additional Supplementary Files [file 41467_2025_60708_MOESM2_ESM.pdf]

Description of Additional Supplementary Files

File Name: Supplementary Data 1

Description: List of codon optimised sequences.

File Name: Supplementary Data 2

Description: List of homologues of eudicot squalene synthase sequences used in gene tree analysis for the relationship of Euphorbia squalene/peplusol synthases in *Euphorbia*.
