# Supplementary material for: Evolution of linear triterpenoid biosynthesis within the Euphorbia genus
Source: Nat Commun. 2025 Jul 1;16:5602. doi: 10.1038/s41467-025-60708-2 (PMC12219392; doi:10.1038/s41467-025-60708-2)
Supplement: Supplementary file 5 — Reporting Summary [file 41467_2025_60708_MOESM5_ESM.pdf]

nature portfolio

Corresponding author(s): Ian A. Graham

Last updated by author(s): 19th May 2025

Reporting Summary

Nature Portfolio wishes to improve the reproducibility of the work that we publish. This form provides structure for consistency and transparency in reporting. For further information on Nature Portfolio policies, see our Editorial Policies and the Editorial Policy Checklist.

Statistics

For all statistical analyses, confirm that the following items are present in the figure legend, table legend, main text, or Methods section.

n/a	Confirmed	
☐	☒	The exact sample size (*n*) for each experimental group/condition, given as a discrete number and unit of measurement
☐	☒	A statement on whether measurements were taken from distinct samples or whether the same sample was measured repeatedly
☐	☒	The statistical test(s) used AND whether they are one- or two-sided<br>*Only common tests should be described solely by name; describe more complex techniques in the Methods section.*
☒	☐	A description of all covariates tested
☒	☐	A description of any assumptions or corrections, such as tests of normality and adjustment for multiple comparisons
☐	☒	A full description of the statistical parameters including central tendency (e.g. means) or other basic estimates (e.g. regression coefficient) AND variation (e.g. standard deviation) or associated estimates of uncertainty (e.g. confidence intervals)
☐	☒	For null hypothesis testing, the test statistic (e.g. *F*, *t*, *r*) with confidence intervals, effect sizes, degrees of freedom and *P* value noted<br>*Give P values as exact values whenever suitable.*
☒	☐	For Bayesian analysis, information on the choice of priors and Markov chain Monte Carlo settings
☒	☐	For hierarchical and complex designs, identification of the appropriate level for tests and full reporting of outcomes
☒	☐	Estimates of effect sizes (e.g. Cohen's *d*, Pearson's *r*), indicating how they were calculated

Our web collection on statistics for biologists contains articles on many of the points above.

Software and code

Policy information about availability of computer code

Data collection	RNA sequencing data were out-sourced and data were provided by service providers. No additional software was used for sequencing data collection.<br>LC-MS data were collected and analysed using the Thermo Xcalibur v 4.0 software. Triterpenoids were identified by comparing exact mass and retention time values to those of authentic standards (either commercial or NMR-validated) run under the same conditions.<br>GC-MS date were collected using Leco ChromaTOF v. 4.50.8.0 software, converted to .raw file and analysed using the Thermo Xcalibur software as above.<br>NMR data collection, analysis and visualization were done using: Bruker Topspin, Bruker Amix and Mestre NMR suite<br>Protein modeling was done using AlphaFold3 server (https://alphafoldserver.com/), with no ligands selected.
Data analysis	Trinity (v2.15.1) was used for E. lateriflora RNA-seq data De novo Assembly.<br>Phylogenetic analysis were done using: Kalign multiple sequence alignment software (https://www.ebi.ac.uk/Tools/msa/kalign) to obtain protein sequence alignment, MUSCLE v3.2 software (for building the alignments), Gblocks v0.9144 software (for generation of conservative alignment blocks by removing highly polymorphic regions), MrBayes v3.2.665 software (for constructing the gene tree), Figtree v1.4.3 (http://tree.bio.ed.ac.uk/software/figtree/) program was used for tree visualization.<br>Protein structural analysis were performed using: ChimeraX version: 1.7 (2023-12-19).

For manuscripts utilizing custom algorithms or software that are central to the research but not yet described in published literature, software must be made available to editors and reviewers. We strongly encourage code deposition in a community repository (e.g. GitHub). See the Nature Portfolio guidelines for submitting code & software for further information.

Data

Policy information about availability of data

All manuscripts must include a data availability statement. This statement should provide the following information, where applicable:

- Accession codes, unique identifiers, or web links for publicly available datasets
- A description of any restrictions on data availability
- For clinical datasets or third party data, please ensure that the statement adheres to our policy

ElSS-L1 and ElSS-L2 CDS sequence data have been deposited in GenBank (gene accessions PP978604 and PP978605). E. lateriflora RNAseq data are available as GenBank BioProject ID PRJNA1131357. All other study data are included in the article and/or supporting information.

Research involving human participants, their data, or biological material

Policy information about studies with human participants or human data. See also policy information about sex, gender (identity/presentation), and sexual orientation and race, ethnicity and racism.

Reporting on sex and gender	not applicable
Reporting on race, ethnicity, or other socially relevant groupings	not applicable
Population characteristics	not applicable
Recruitment	not applicable
Ethics oversight	not applicable

Note that full information on the approval of the study protocol must also be provided in the manuscript.

Field-specific reporting

Please select the one below that is the best fit for your research. If you are not sure, read the appropriate sections before making your selection.

☒ Life sciences ☐ Behavioural & social sciences ☐ Ecological, evolutionary & environmental sciences

For a reference copy of the document with all sections, see nature.com/documents/nr-reporting-summary-flat.pdf

Life sciences study design

All studies must disclose on these points even when the disclosure is negative.

Sample size	No sample size calculations were performed. Maximum number of biological replications were chosen based on the availability of the biological material and space to perform experiments. A biological sample size of minimum 3 was also chosen to meet journal requirements. We have observed generally a very low biological variation, when using number of replications listed above. We have therefore concluded there was no need for increasing sample size.
Data exclusions	No data were excluded from the analysis.
Replication	No replication was needed for the RNA-Seq analysis as the data was used qualitatively to obtain gene sequences rather than quantitatively. Transient gene expression in N. benthamiana was performed using four or five biological replicates for each experimental group (four or five plants infiltrated for each combination of constructs). Metabolite profiling of latex from the three Euphorbia species was performed using four biological replicates. Gene expression studies in S. cerevisiae was performed using three independent transformants for each of the constructs/background strains analyzed. In vitro experimentation on enzyme activity did not involve replication of individual assays and the data is presented as semi-quantitative.
Randomization	Not applicable, no formal randomization was carried out. Randomization was not required for the metabolite analysis carried out.
Blinding	Not applicable, no blinding experiments were carried out for metabolite experiments as there would be no opportunity for operator actions to bias the outcomes.

Reporting for specific materials, systems and methods

We require information from authors about some types of materials, experimental systems and methods used in many studies. Here, indicate whether each material, system or method listed is relevant to your study. If you are not sure if a list item applies to your research, read the appropriate section before selecting a response.

Materials & experimental systems

n/a	Involved in the study
☒	☐ Antibodies
☒	☐ Eukaryotic cell lines
☒	☐ Palaeontology and archaeology
☒	☐ Animals and other organisms
☒	☐ Clinical data
☒	☐ Dual use research of concern
☐	☒ Plants

Methods

n/a	Involved in the study
☒	☐ ChIP-seq
☒	☐ Flow cytometry
☒	☐ MRI-based neuroimaging

Dual use research of concern

Policy information about dual use research of concern

Hazards

Could the accidental, deliberate or reckless misuse of agents or technologies generated in the work, or the application of information presented in the manuscript, pose a threat to:

No	Yes
☒	☐ Public health
☒	☐ National security
☒	☐ Crops and/or livestock
☒	☐ Ecosystems
☒	☐ Any other significant area

Experiments of concern

Does the work involve any of these experiments of concern:

No	Yes
☒	☐ Demonstrate how to render a vaccine ineffective
☒	☐ Confer resistance to therapeutically useful antibiotics or antiviral agents
☒	☐ Enhance the virulence of a pathogen or render a nonpathogen virulent
☒	☐ Increase transmissibility of a pathogen
☒	☐ Alter the host range of a pathogen
☒	☐ Enable evasion of diagnostic/detection modalities
☒	☐ Enable the weaponization of a biological agent or toxin
☒	☐ Any other potentially harmful combination of experiments and agents

Plants

Seed stocks	Euphorbia peplus seeds were obtained from All Rare Herbs (Australia). Euphorbia lateriflora cuttings were obtained from Botanic Garden Meise, Belgium (specimen GH-0-BR-2014067883, collected south of Oduponkpeke, Accra Plains, Ghana). Euphorbia lathyris plants were collected from the wild (Heslington, York, UK, location N53.94292547361107, E-1.0491734918701132)
Novel plant genotypes	No novel plant genotypes, including transgenic lines, were produced for this manuscript.
Authentication	Euphorbia lathyris plants were authenticated using DNA sequencing of a conserved ortholog set of genes as cited in the mansucript using methodology detailed in the methods section of the manuscript.
